# Stabilization and attributive amelioration of sugarcane juice by naturally derived preservatives using aonla and moringa extract

**DOI:** 10.1002/fsn3.2262

**Published:** 2021-03-27

**Authors:** Moazzam Rafiq Khan, Ayesha Syed, Sania Zia, Waqar Ahmed, Rana Muhammad Aadil, Muhammad Faisal Manzoor, Muhammad Inam‐Ur‐Raheem, Muhammad Abid, Muhammad Asim Shabbir, Shahnah Qureshi, Ahmad Din, Emaad Karrar

**Affiliations:** ^1^ National Institute of Food Science and Technology University of Agriculture Faisalabad Pakistan; ^2^ School of Food and Biological Engineering Jiangsu University Zhenjiang China; ^3^ Institute of Food and Nutritional Sciences Pir Mehr Ali Shah Arid Agriculture University Rawalpindi Pakistan; ^4^ Department of Food Engineering and Technology Faculty of Engineering and Technology University of Gezira Wad Medani Sudan

**Keywords:** aonla, browning, moringa, natural preservatives, sugarcane juice

## Abstract

Sugarcane juice (SCJ) is a cheap, popular, and very nutritious beverage served at roadside stalls in many countries during harvesting season. The juice is normally consumed immediately after extraction as fermentation sets within a few hours of extraction. Preserving the raw sugarcane juice is always challenging because it spoils within a few hours of extraction due to fermentation. Therefore, the bottling, distribution, and marketing of sugarcane are difficult tasks. The present study was designed to investigate the effect of naturally derived preservatives using aonla extract (AE) and moringa extract (ME) in different proportions (0%, 3%, 5%, and 7%) for the preservation of SCJ during 21 days of the storage period at 4 ± 2°C temperature. The effect of extracts and storage time were analyzed on physicochemical parameters, bioactive compounds, enzymatic, microbiological, and sensory analyses of SCJ. A significant improvement in pH of 5% AE (5.30 ± 0.06) and 5% ME (5.36 ± 0.02) was observed at 21 days as compared to control (5.89 ± 0.02). The total phenolic contents in 7% ME were also observed to be retained (4.4 ± 0.02 mg GAE/mL) at 21 days as compared to control (2.65 ± 0.03 mg GAE/mL). Other physicochemical and phytochemical analyses including titratable acidity, total soluble solids, total flavonoids, ascorbic acid, 2,2‐Diphenyl‐2‐picrylhydrazyl (DPPH), and ferric reducing antioxidant power assay (FRAP) also indicated that SCJ treated with ME was significantly stable (*p* < .05) regarding quality parameters, nutritional and sensory attributes at different storage intervals. These findings may be practical for the large‐scale production, storage, and marketing of SCJ products.

## Graphical abstract

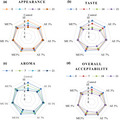

## 1 INTRODUCTION

Sugarcane juice (SCJ) is a ready‐to‐serve drink traditionally consumed as a beverage all over the world. SCJ is competing with other soft drinks in the market due to its refreshing sweet taste, due to which sugarcane farming and harvesting are increasing with time. SCJ is rich in sucrose, polyphenolic compounds, minerals (such as potassium, calcium, sodium, iron, and magnesium), ascorbic acid, pigments, and fine bagasse (Kaavya et al., 2019). The SCJ is prepared from sugarcane stalks, which is first washed, and juice is extracted by crushing the stalks between a mechanical roller crusher and consumed immediately. SCJ marketing and processing operations are quite a few because sugarcane fermentation begins immediately the following extraction as it contains 80% water and 20% total soluble solids (TSS) with a pH greater than 4.6 and water activity 0.99_aW_ (Abhilasha & Pal, 2018). The presence of carotenoids, anthocyanins, melanoidins, flavonoids, melanin, and substances from the alkaline oxidation of fructose in varying quantities in sugarcane affects its color, which consequently affects consumer appeal (Bomdespacho et al., 2018). Enzymatic browning is a significant contributor to SCJ degradation. During processing, mechanical stress causes cellular delocalization of enzymes and their substrates, resulting in biochemical deteriorations including enzymatic browning, off‐flavor growth, and texture collapse. Enzymatic browning is normally produced by the enzyme polyphenol oxidase (PPO, EC 1.10.3.1), which transforms phenolic compounds into dark‐colored pigments in the presence of oxygen. Other enzymes, such as peroxidase (POD, EC 1.11.1.7), may also play a significant role in total browning (Hithamani et al., 2018). These are the main hurdles in the industrial production of SCJ, thereby limiting the earning opportunities of its manufacturing and marketing (Zia et al., 2019; Manzoor, Ahmad, Ahmed, Siddique, et al., 2020). Therefore, the bottling, distribution, and marketing of fresh SCJ are difficult tasks.

The fermentation process must be controlled to preserve the SCJ by applying some processing and preservation techniques (Garud et al., 2018). Researchers and food industries have studies different preservation technologies such as pasteurization, pulsed electric field, ultrasound, microwave, and high‐pressure processing to secure enzymatic reactions and microbial growth in fresh SCJ. Although appropriate use of such technologies makes it possible to extend the distribution area from field to consumers and also decreases the transportation cost of the supply chain, these advanced technologies increase the overall operating and processing cost (Ahmed et al., 2020; Kaavya et al., 2019; Manzoor, Ahmed, Ahmad, Aadil, et al., 2020; Manzoor et al., 2019). Today the food industry is more concerned with the product shelf‐life enhancement and food safety for which chemical preservatives are increasingly used. These chemical preservatives are artificially synthesized, for example, sodium and potassium salts of lactic acid, benzoic acid, and sodium diacetate, etc. Most of these chemical preservatives are expensive and pose health problems as they are carcinogenic and cause digestive and respiratory problems (Ramachandran et al., 2017).

Nowadays, in the health‐conscious consumer market, food manufacturers demand natural, safe, and environmentally friendly food preservatives that are less expensive, more nutritious, and can be easily obtained (Aadil et al., 2018, 2015; Arshad et al., 2021). Natural preservatives based on fruits, herbs, spice, and flavoring oils have shown feasibility in various fruit juices (Bouarab Chibane et al., 2019). In recent years, the extract obtained from natural sources such as *Emblica officinalis* and *Moringa oleifera* has been gaining more popularity (Mehwish et al., 2021) as a preservative agent in food and soft drinks. *M. oleifera* leaves are the rich source of potent nutrients and phytochemical components that allow these leaves to be utilized as a natural preservative in the food industry and as an alternative to the chemical preservative in future. The leave extract rich in phytochemicals such as phenolic, flavonoids, alkaloids, saponins, tannins, terpenoids, glycosides, and other polyphenol compounds provide an antimicrobial preservative effect (Busani et al., 2011). Aonla fruit, Indian gooseberry, is a highly nutritive fruit having great medicinal properties and possesses the highest level of heat‐stable vitamin C. Aonla is presently underutilized fruit because of its high acidity and astringency but has enormous potential in the world market. A lot of research has been reported on the utilization of aonla fruit in the formulation of various products (Karpagavalli et al., 2014), but still, there is a lot of scopes to explore the possibility of its utilization as a preservative agent in the beverage market. Clinical in vivo and in vitro assays show that the aonla fruit juice extracts have potential antioxidant, antimicrobial and anti‐inflammatory properties due to the presence of ascorbic acid, phenols, flavonoids, tannins, and polyphenols (Kumari & Khatkar, 2019).

This study aims to enhance the shelf‐life of SCJ using naturally derived preservatives. No study has been performed to investigate the effect of moringa leaves and aonla fruit extract as a preservative for preserving SCJ in the refrigerator for up to 21 days. Therefore, this research study was designed to investigate the effect of different levels of *M. oleifera* leaves and aonla fruit (*Emblica officinalis*) extract and storage on physicochemical parameters, bioactive compounds, antioxidant and enzymatic activity, and microbiological analysis of SCJ.

## 2 METHODOLOGY

The proposed study was carried out at Fruits & Vegetable Laboratory, National Institute of Food Science and Technology, University of Agriculture, Faisalabad, Pakistan.

### 2.1 Procurement of raw material

Fresh, healthy, and disease‐free stems of sugarcane and aonla were procured from Ayyub Agriculture Research Institute, Faisalabad, Pakistan. Moringa leaves were collected from the agronomy fields of the University of Agriculture, Faisalabad, Pakistan. Aonla fruit was purchased from the local market of Faisalabad, Pakistan. All the raw material was brought to the laboratory for further analysis.

### 2.2 Preparation of SCJ, aonla, and moringa extracts

SCJ was prepared by following the process of Zia et al. (2019). Fresh aonla fruit and moringa leaves were washed with distilled water to remove dust and dried in a cabinet dryer (Harvest saver Model # R‐54, Commercial Dehydrator System Inc. Eugene, United States) at 60℃ and ground to a fine powder in a mechanical blender (GEKA‐283, Westpoint, Lahore). 15 g of each powder were mixed individually with 200 ml of ethanol (90% for aonla and 70% for moringa) and maintained on a magnetic agitator with constant stirring (200 RPM) for 30 min at room temperature. The mixtures were centrifuged at 3,500 RPM for about 25 min at 15℃. The supernatant was taken and stored overnight at 4.0 ± 2℃ and then filtered through filter paper. The filtrates were concentrated to 50% initial volume at 50℃ using a rotary vacuum evaporator (VP30, LabTech, UK) and stored at −20℃ for further use.

### 2.3 Preparation of SCJ and extracts blend

The treatments for the preparation of SCJ and extracts blend are shown in Table 1. All the treatments were stored in airtight sterilized glass bottles and kept at 4 ± 2℃ for 21 days. All the treatments were subjected to analytical trials in three replicates.

**TABLE 1:** Different treatment levels of aonla and moringa extract

Treatments	
Control	100% SCJ (No addition of extract)
AE 3%	3% of Aonla extract in 97% of SCJ
AE 5%	5% of Aonla extract in 95% of SCJ
AE 7%	7% of Aonla extract in 93% of SCJ
ME 3%	3% of moringa extract in 97% of SCJ
ME 5%	5% of moringa extract in 95% of SCJ
ME 7%	7% of moringa extract in 93% of SCJ

### 2.4 Analysis of naturally preserved SCJ during storage

#### 2.4.1 Determination of pH, titratable acidity, and total soluble solids (TSS)

The pH (AOAC Method No. 981.21), titratable acidity (AOAC Method No. 942.15), and TSS (IS 13815:2010. ISO2173: 200) were determined by following the method of AOAC (2005) and Aadil et al. (2013).

#### 2.4.2 Determination of total phenolic, flavonoid and ascorbic acid content (TPC, TFC and AA)

The total phenolic contents were determined using the Folin–Ciocalteau (FC) reagent method as stated by Sreeramulu et al. (2013), and the results were recorded as mg gallic acid equivalent (GAE)/ml of the SCJ. The TFC was measured using the colorimetric aluminum chloride method as described by Mishra et al. (2011), and the findings were stated as mg catechin equivalents (CE)/mL of SCJ. AA values were determined using the protocol described by Silva (2005) and the findings were reported as mg ascorbic acid (AA)/100 ml of SCJ.

#### 2.4.3 Free radical scavenging activity (DPPH)

The antioxidant activity to scavenge free radicals was measured by following the procedure of Manzoor, Zeng, et al. (2020). This method is based on the ability of the antioxidant to scavenge the 2, 2 Diphenyl‐2‐picrylhydrazyl (DPPH) cation radicals. DPPH is an oxidizing radical which is highly stable and reacts with free hydrogen atoms from antioxidant resulting in the formation of yellow color hydrazine. The free radical scavenging activity was measured for absorbance at 517nm expressed as Trolox and ascorbic acid equivalent and results were calculated in percentage of DPPH by using the formula below:

$$\text{Antiradical activity (}\%)=\frac{\text{Absorbance}\;\left(\text{control}\right)‐\text{Absorbance}\;\left(\text{sample}\right)}{\text{Absorbance}\;\left(\text{control}\right)}\times 100.$$

#### 2.4.4 Ferric reducing antioxidant power assay (FRAP)

The antioxidant capacity of SCJ to reduce ferric tripyridyltriazine into blue color was measured by following the procedure of Thaipong et al. (2006). Trolox was used as standard and the results were expressed as µg Trolox equivalent per mL of SCJ sample.

#### 2.4.5 Determination of enzymatic activity

Enzymatic activity of SCJ samples was observed by following the procedure of Kunitake et al. (2014). The SCJ sample was incubated with hydrogen peroxide, phosphate buffer (pH 5.0), and an alcoholic solution of peroxidase for 15 min at 30°C for the peroxidase enzyme activity. The reaction was then terminated with sodium metabisulfate and the absorbance was measured at 470 nm on a spectrophotometer. The SCJ sample was incubated with phosphate buffer (pH 6.9) and catechol for 30 min at 30℃ to determine the polyphenol oxidase activity. The reaction was then terminated with perchloric acid and the absorbance was measured at 395 nm on a spectrophotometer. A blank absorbance of SCJ with only phosphate buffer was determined. The enzymatic activity was expressed in U/mL with one‐unit equivalent to a variation of 0.001 absorbances per min per mL of the SCJ sample.

Enzymatic activity was calculated by using calculations

$$\text{Enzymatic activity}\;(\mu \text{/ml)}=\frac{\text{Absorbance}\left(\text{sample}\right)‐\text{Absorbance}\left(\text{blank}\right)}{0.001\times t}$$

Where t is the incubation time of the sample with reagents (min).

#### 2.4.6 Microbiological analysis

The microbiological analysis was carried out as per the protocol mentioned by Pérez‐Grijalva et al. (2018). The aerobic mesophiles were measured using the standard pour plate method. The serial dilution with 0.85% normal saline solution was made for SCJ samples and appropriate plates were incubated at 37 ± 1°C for 48 hr. Yeast and mold count was made on potato dextrose agar plates by incubating a 25 ± 1°C for 120 hr. After incubation, the colony‐forming units were counted by multiplying the dilution factor to obtain total plate count, and yeast and mold count.

#### 2.4.7 Sensory analysis

Sensory analysis was performed by following the procedure as stated by Huang et al. (2015). The taste of 20 panelists was screened using a synthetic solution having a mixture of sucrose (7–14 ºBrix) and malic acid as well as a whole fruit in the prescreening session. From 20 panelists, 10 were selected, which had better sensory reception. These panelists were allowed to evaluate the SCJ sample for taste, flavor, mouthfeel, and overall acceptability on a hedonic scale of 1 to 9 points (1 point shows the lowest and 9 points shows the highest level of acceptance).

### 2.5 Statistical analysis

The findings were expressed as mean ± standard deviation (*SD*) of triplicates. The software Statistics 10 was used for the complete analysis of variance of data using two‐way factorial design and treatment means were compared at the level of significance (*p* < .05).

## 3 RESULTS AND DISCUSSION

### 3.1 Effect on pH, titratable acidity, and TSS (ºBrix)

The results regarding pH, titratable acidity, and TSS (ºBrix) of control (untreated) and treated SCJ samples with different levels of natural extracts (AE and ME) are shown in Table 2. Statistically, both treatments and storage had a significant effect (*p* < .05) on pH and titratable acidity of naturally preserved SCJ as compared to control while nonsignificant effect (*p* < .05) on TSS. TSS remained stable for all the treatments and was not affected by storage time (21 days). Moreover, it was observed from the mean values that treatments with high inclusion levels of AE showed a greater drop in pH and rise in acidity while the least was observed in ME incorporated SCJ at zero days of storage. This may be due to the presence of high ascorbic acid content in AE (Goraya & Bajwa, 2015). However, a higher increase in pH and decrease in acidity was found in ME incorporated SCJ as compared to AE incorporated SCJ, with the increase in the storage period. Minimum mean pH and maximum mean acidity were observed at zero days of storage while maximum mean pH and minimum mean acidity were recorded after 21 days of storage. This increase in pH and decrease in acidity might be due to the acidic hydrolysis of nonreducing sugars and converted to reducing sugars with the increase in storage time (Zia et al., 2019). The result regarding the increase in pH and decrease in acidity is by the storage study of guava whey juice fortified with *M. oleifera* aqueous extract to increase its shelf life (Hashemi et al., 2018b). Ajayi et al. (2019) reported a decrease in acidity and an increase in pH in zobo juice preserved with moringa leaves and ginger extracts at different storage conditions. Similarly, a decrease in acidity and an increase in pH were recorded in ready to serve beverage prepared from aloe vera gel and aonla fruit juice extract with the increase in storage time (Sasikumar, 2015). In the present study, it was found that AE and ME up to 5% are more effective in preserving the pH and acidity of SCJ as compared to control and other treatments during storage.

**TABLE 2:** Effect of naturally derived preservatives and storage time on the pH, titratable acidity and ºBrix of SCJ

Quality attribute	Treatments	Storage intervals (Days)			
		0	7	14	21
pH	Control	5.42 ± 0.04 cd	5.50 ± 0.05 c	5.72 ± 0.01 b	5.89 ± 0.02 a
	AE 3%	5.31 ± 0.03 cd	5.38 ± 0.02 c	5.46 ± 0.02 b	5.58 ± 0.03 a
	AE 5%	5.23 ± 0.1 b	5.24 ± 0.01b	5.27 ± 0.01 ab	5.30 ± 0.06 a
	AE 7%	5.16 ± 0.02 bc	5.19 ± 0.04 c	5.26 ± 0.04 b	5.34 ± 0.05 a
	ME 3%	5.36 ± 0.06 c	5.43 ± 0.05 bc	5.52 ± 0.02 b	5.69 ± 0.03 a
	ME 5%	5.26 ± 0.03 b	5.28 ± 0.03 b	5.31 ± 0.03 b	5.36 ± 0.02 a
	ME 7%	5.19 ± 0.02 cd	5.23 ± 0.02 c	5.31 ± 0.01 b	5.41 ± 0.01 a
Titratable acidity (%)	Control	0.96 ± 0.06 a	0.89 ± 0.04 b	0.81 ± 0.01 c	0.72 ± 0.03 d
	AE 3%	1.03 ± 0.05 a	1.07 ± 0.02 ab	1.09 ± 0.03 b	1.11 ± 0.04 bc
	AE 5%	1.07 ± 0.05 a	1.13 ± 0.01 b	1.18 ± 0.04 bc	1.22 ± 0.02 c
	AE 7%	1.10 ± 0.02 a	1.12 ± 0.05 b	1.15 ± 0.02 bc	1.20 ± 0.01 bc
	ME 3%	0.89 ± 0.01 a	0.96 ± 0.02 ab	1.03 ± 0.03 b	1.11 ± 0.02 bc
	ME 5%	0.87 ± 0.03 a	0.94 ± 0.02 ab	1.04 ± 0.05 b	1.08 ± 0.03 c
	ME 7%	0.83 ± 0.02 a	0.88 ± 0.04 b	0.94 ± 0.01 bc	1.04 ± 0.03 c
Total soluble solids (ºBrix)	Control	20.54 ± 0.02 a	20.49 ± 0.03 a	20.44 ± 0.03 a	20.34 ± 0.02 a
	AE 3%	20.48 ± 0.05 a	20.45 ± 0.02 a	20.41 ± 0.04 a	20.37 ± 0.01 a
	AE 5%	20.50 ± 0.05 a	20.47 ± 0.06 a	20.43 ± 0.02 a	20.39 ± 0.04 a
	AE 7%	20.51 ± 0.03 a	20.50 ± 0.01 a	20.48 ± 0.02 a	20.46 ± 0.03 a
	ME 3%	20.51 ± 0.01 a	20.48 ± 0.04 a	20.45 ± 0.01 a	20.40 ± 0.01 a
	ME 5%	20.52 ± 0.03 a	20.52 ± 0.02 a	20.51 ± 0.01 a	20.48 ± 0.02 a
	ME 7%	20.54 ± 0.05 a	20.53 ± 0.03 a	20.49 ± 0.03 a	20.45 ± 0.03 a

Note

Mean value ± *SD* of triplicates. Figures in the same row with different letters are significantly different (*p* < .05).

### 3.2 Effect on the total phenolic and flavonoid content (TPC and TFC)

The changes in the mean values of TPC and TFC of control (untreated) and treated SCJ samples after 21 days of storage are shown in Table 3. The mean values of TPC and TFC of all samples revealed a significant decrease (*p* < .05) after 21 days of storage period. The reduction level in TPC and TFC was significantly higher (*p* < .05) in control and all AE incorporated SCJ treatments as compared to ME incorporated SCJ treatments. The oxidative degradation of phenolic compounds and polymerization with protein during storage may be the reason for a decrease in TPC and TFC (Huang et al., 2015). Another reason may be that polyphenols are involved in some specific physicochemical interactions with the soluble solids especially with the solids of the cell wall (Karpagavalli et al., 2014).

**TABLE 3:** Effect of naturally derived preservatives and storage period on TPC, TFC, AA content and antioxidant activity (DPPH and FRAP) of SCJ

Quality attribute	Treatments	Storage intervals (Days)			
		0	7	14	21
Total phenolic content (mg GAE/ml)	Control	3.96 ± 0.02 a	3.65 ± 0.04 b	3.38 ± 0.02 c	2.65 ± 0.03 d
	AE 3%	4.10 ± 0.03 a	4.06 ± 0.03 b	3.98 ± 0.03 bc	3.78 ± 0.04 c
	AE 5%	4.32 ± 0.01 a	4.28 ± 0.06 a	4.22 ± 0.05 ab	4.15 ± 0.01 b
	AE 7%	4.45 ± 0.05 a	4.42 ± 0.01 a	4.37 ± 0.03 ab	4.25 ± 0.02 c
	ME 3%	4.32 ± 0.02 a	4.28 ± 0.04 ab	4.22 ± 0.02 b	4.10 ± 0.03 c
	ME 5%	4.46 ± 0.03 a	4.46 ± 0.03 a	4.44 ± 0.05 a	4.4 ± 0.02 b
	ME 7%	4.68 ± 0.01 a	4.66 ± 0.01 a	4.63 ± 0.03 ab	4.51 ± 0.05 b
Total flavonoids compounds (mg CE/ml)	Control	3.52 ± 0.03 a	3.47 ± 0.04 b	3.36 ± 0.03 c	3.29 ± 0.04 d
	AE 3%	3.62 ± 0.01 a	3.56 ± 0.03 ab	3.48 ± 0.04 b	3.39 ± 0.03 c
	AE 5%	3.69 ± 0.04 a	3.69 ± 0.01 a	3.69 ± 0.01 a	3.62 ± 0.05 ab
	AE 7%	3.76 ± 0.02 a	3.74 ± 0.04 a	3.69 ± 0.02 b	3.64 ± 0.01 bc
	ME 3%	3.74 ± 0.03 a	3.69 ± 0.03 ab	3.62 ± 0.05 b	3.54 ± 0.02 c
	ME 5%	3.81 ± 0.05 a	3.80 ± 0.01 a	3.77 ± 0.06 a	3.71 ± 0.04 ab
	ME 7%	3.91 ± 0.01 a	3.90 ± 0.03 a	3.87 ± 0.03 ab	3.76 ± 0.03 b
Ascorbic acid (mg/100 ml)	Control	0.61 ± 0.02 a	0.56 ± 0.02 b	0.44 ± 0.03 c	0.31 ± 0.03 d
	AE 3%	0.84 ± 0.04 a	0.78 ± 0.03 ab	0.67 ± 0.01 c	0.55 ± 0.01 d
	AE 5%	0.92 ± 0.01 a	0.88 ± 0.01 a	0.83 ± 0.05 ab	0.76 ± 0.05 b
	AE 7%	1.04 ± 0.03 a	0.99 ± 0.05 a	0.89 ± 0.02 b	0.8 ± 0.033 bc
	ME 3%	0.73 ± 0.04 a	0.69 ± 0.02 ab	0.58 ± 0.03 b	0.47 ± 0.03 c
	ME 5%	0.79 ± 0.02 a	0.78 ± 0.04 a	0.74 ± 0.04 ab	0.69 ± 0.02 b
	ME 7%	0.86 ± 0.03 a	0.83 ± 0.01 a	0.76 ± 0.01 ab	0.69 ± 0.01 b
DPPH (%)	Control	78.38 ± 0.04 a	74.23 ± 0.01 ab	67.82 ± 0.03 b	57.89 ± 0.05 c
	AE 3%	86.67 ± 0.02 a	82.34 ± 0.01 ab	77.45 ± 0.02 b	66.04 ± 0.03 c
	AE 5%	91.45 ± 0.05 a	89.23 ± 0.04 a	86.56 ± 0.01 ab	82.45 ± 0.03 b
	AE 7%	95.23 ± 0.01 a	92.98 ± 0.06 a	87.67 ± 0.03 ab	81.56 ± 0.04 b
	ME 3%	89.78 ± 0.02 a	83.56 ± 0.03 ab	78.98 ± 0.05 ab	70.51 ± 0.03 b
	ME 5%	93.31 ± 0.06 a	92.56 ± 0.01 a	89.34 ± 0.04 a	85.08 ± 0.04 ab
	ME 7%	96.45 ± 0.03 a	93.27 ± 0.05 ab	90.13 ± 0.02 ab	84.87 ± 0.01 b
FRAP (%)	Control	77.12 ± 0.05 a	73.98 ± 0.03 ab	66.12 ± 0.01 b	56.12 ± 0.02 c
	AE 3%	83.23 ± 0.03 a	81.02 ± 0.02 ab	75.34 ± 0.07 b	67.34 ± 0.05 bc
	AE 5%	86.23 ± 0.01 a	84.13 ± 0.05 a	82.34 ± 0.02 a	79.23 ± 0.02 ab
	AE 7%	90.34 ± 0.03 a	86.45 ± 0.01 a	81.45 ± 0.04 ab	78.67 ± 0.01 ab
	ME 3%	84.12 ± 0.02 a	81.23 ± 0.04 ab	76.89 ± 0.01 b	68.78 ± 0.03 bc
	ME 5%	88.78 ± 0.03 a	86.56 ± 0.05 a	83.45 ± 0.03 ab	79.67 ± 0.01 b
	ME 7%	93.64 ± 0.02 a	89.13 ± 0.01 a	85.34 ± 0.05 ab	79.01 ± 0.04 b

Note

Mean value ± *SD* of triplicates. Values in the same row with different letters are significantly different (p < .05).

All treatments of ME incorporated SCJ revealed less reduction in TPC and TFC as compared to control, which showed that ME treatments give better stability during storage. However, the SCJ sample that was contained 5% ME was significantly (*p* < .05) stable and considered to have higher TPC (4.4 ± 0.02 mg GAE/ml) and TFC (3.71 ± 0.04 mg CE/ml) with the increase in storage period. The significant stability could be due to the inactivation of the enzyme that utilizes these compounds as substrate and cause the degradation during storage. The results regarding the ME (5%) incorporated SCJ were similar to the finding reported in the 10 days storage study of guava whey beverage fortified with different levels (2.5%, 5%, and 7.5%) of *M. oleifera* leave extracts (Ali et al., 2015). Hashemi et al. (2018a) have also reported a slight decrease in TPC during one month of storage of fresh sweet orange juice preserved with 10% of moringa leave extract, 10% ginger, and 10% beetroot juice as compared to other treatments. Current results concluded that SCJ treatments with moringa leave extracts had a positive effect on TPC and TFC in comparison to control and AE treated SCJ samples.

### 3.3 Effect on ascorbic acid (AA) content

The effect of the treatments on AA content is shown in Table 3. All the treatments showed a significant decrease (*p* < .05) in AA content during the 21 days of storage time. However, it was observed that AE incorporated SCJ retained more ascorbic acid as compared to control and ME during 21 days of storage. This might be due to the presence of the higher AA content in AE and their synergistic effect, which is more effective to reduce the oxidation process and enzymatic activity (Meena et al., 2017). Similar results were also reported by Yadav et al. (2014) on 90 days of storage of carrot juice blended with aonla, pomegranate, and grape juice at the level of 15% each. Moreover, the maximum AA was observed in the SCJ sample treated with 5% of AE as AE contained the highest AA content. The findings confirm with the research study of Bhardwaj and Mukherjee (2011) who reported the preservation of the Kinnow juice by blending of aonla and ginger extracts (5%).

The decrease in the AA content in control and ME‐treated SCJ was probably because ascorbic acid is sensitive to light, oxygen, and temperature fluctuations. AA was easily oxidized in the presence of oxygen by both enzymatic and nonenzymatic catalysts. The results are by the findings of Ajayi et al. (2019) who reported a decrease in AA content during the 8 weeks storage of zobo juice preserved with moringa leave and ginger extracts at the level of 0.5% and 1% each. Similarly, a decrease in AA content was reported during the storage of mixed fruit and vegetable juice preserved with *M. oleifera* leave extract at the level of 40%, 50%, and 60% (Hashemi et al. 2018b). Hashemi et al. (2018a) also reported the loss of AA in freshly sweet orange juice treated with 10% and 20% of *M. oleifera* leave extract during one‐month storage.

### 3.4 Antioxidant activity

The results regarding the antioxidant activity of control and treated SCJ samples are shown in Table 2. It was revealed that the changes recorded in the free radical scavenging activity (DPPH) and antioxidant capacity (FRAP) were nonsignificant (*p* < .05) during the first 14 days of storage, but the antioxidant activity was significantly reduced (*p* < .05) after 21 days of storage. The level of reduction in antioxidant capacities was more significant (*p* < .05) in control and 3% treatments of AE (66.04 ± 0.03% at 21 days) and ME (70.51 ± 0.03% at 21 days) incorporated SCJ as compared to other treatments. This decrease in antioxidant activity might be due to the loss of phenols, flavonoids, and polyphenolic compounds that contribute to the antioxidant potential of the juice. These losses of phytochemicals might have occurred due to the oxidation and enzyme activities. This decrease in free radical scavenging activity of all treatments with the increase in storage time is by the findings of Hashemi et al. (2018b) who reported a decrease in DPPH value in guava whey juice fortified 1.5% and 2% of *M. oleifera* aqueous extract during two months of storage. The ME at the level of 5% and 7% showed a higher scavenging activity and antioxidant capacity in SCJ during storage. Similar results have been reported by Ali et al. (2015) who studied that ME at the level of 5% and 7.5% show a higher scavenging activity in the guava whey beverage during 10 days of storage.

### 3.5 Enzymatic activity

**FIGURE 1:**
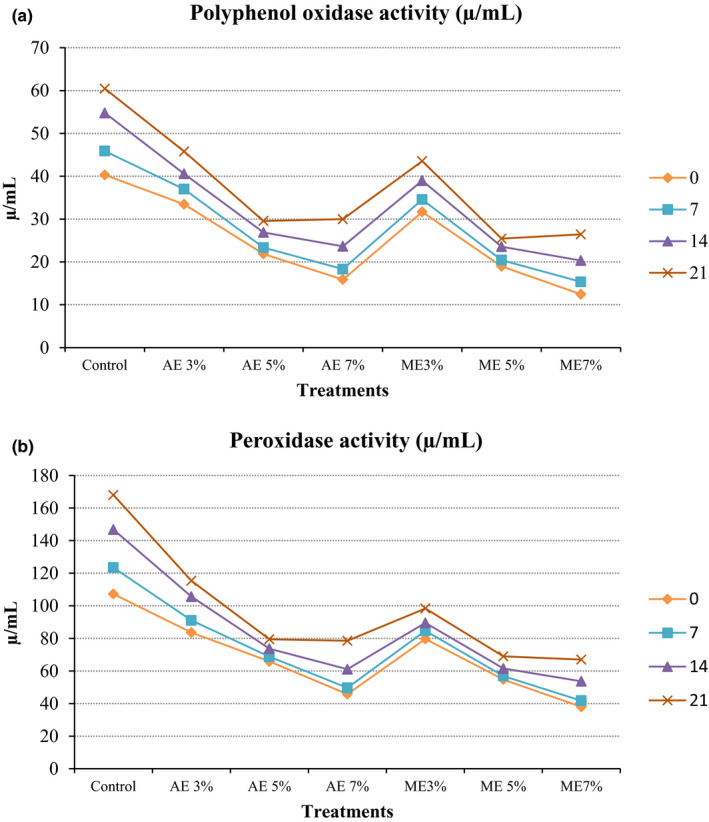
Effect of naturally derived preservatives and storage period on the polyphenol oxidase (a) and peroxidase (b) activity of SCJ

The results regarding enzymatic activity are presented in Figure 1(a,b). It was observed from the mean values that the different levels of the extract significantly (*p* < .05) affect the activity of polyphenol oxidase (PPO) and peroxidase (POD) in SCJ. However, a significant increase (*p* < .05) in enzymatic activity was observed in control, and the 3% level of both extracts incorporated SCJ treatments during storage. This increased activity caused the browning of SCJ during storage, which might be due to oxidation of phenolic compounds, degradation of chlorophyll, and the reaction of an organic acid with sugars that lead to the formation of insoluble browning pigments such as melanin (Meena et al., 2017). The minimum increase in enzymatic browning was observed in the 5% and 7% levels of both extracts incorporated treatments. This could be due to the inactivation of both enzymes (PPO and POD) by the higher concentration of phenolic and ascorbic acid content in higher levels 5% and 7% of both extracts. The results are similar to the finding of Bhardwaj and Mukherjee (2011) who reported a minimum increase in enzymatic activity in kinnow juice blended with 5% of aonla and 3% of ginger juice for six months storage. Bhattacherjee et al. (2013) also reported an increase in enzymatic activity in aonla juice extracted from fruit preserved by steeping in water after 30 days storage.

### 3.6 Microbiological activity

**FIGURE 2:**
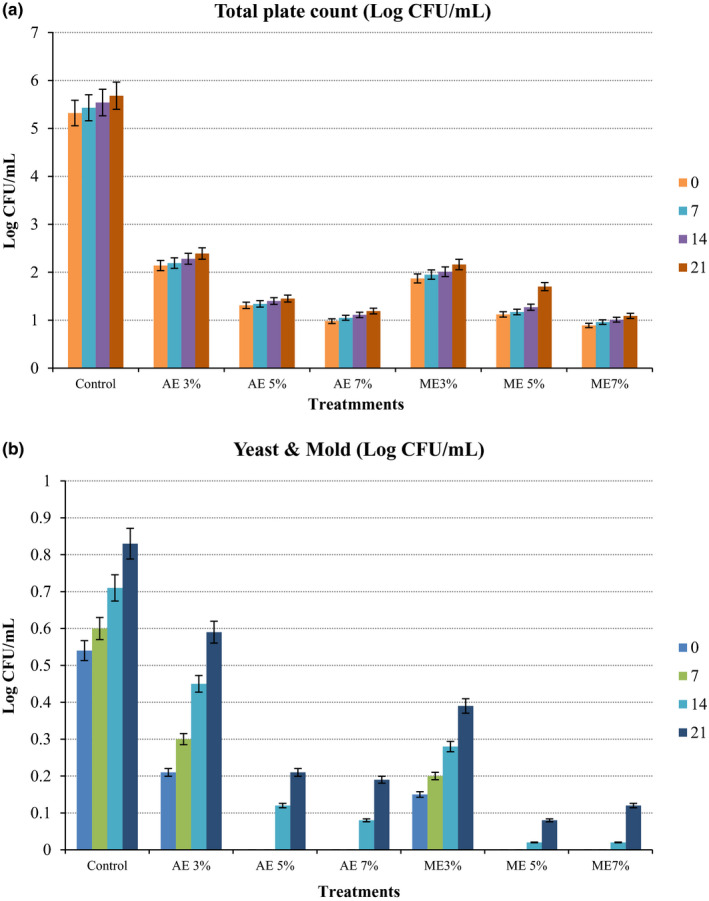
Effect of naturally derived preservatives and storage period on Total plate count (a) and Yeast & Mold (b) of SCJ

The change in the microbiological load of control and treated sugarcane juice during the storage period is shown in Figure 2(a,b). At zero days storage, a significant reduction (*p* < .05) in the total microbial count was observed in all the treatments in comparison with control. But, a gradual increase in microbial growth was shown in all treatments during the storage period. The maximum microbial load was observed in control and the 3% level of both extracts incorporated SCJ treatments after 21 days of storage as compared to other treatments. The 7% level of both AE and ME incorporated SCJ showed the minimum growth of aerobic microbes with an increase in storage time. However, an increase in yeast and mold count was observed which might be due to the inhibitory effect of extracts toward the aerobic microbes and favorable condition toward the growth of yeast and mold (Gajera, 2017). Similarly, Sangeeta and Khatkar (2013) have reported a lesser increase in microbial count in SCJ having a higher proportion of aonla juice which may be due to the preservative effect of ascorbic acid. The results of ME treatments are similar to the findings of Boniface et al. (2020), who reported that 30% of ME was more effective in reducing the aerobic mesophiles, yeast, and fungi count of SCJ as compared to 10 and 20% of moringa leave extracts during 28 days of storage. This increase in the antimicrobial activity of extracts might be due to the microbial inhibition and increased preservation activity of hydrocarbons, alcoholic, and phenolic compounds of MEs. The present research results are similar to the finding of Hashemi et al. (2018b) in the preservation of guava whey juice fortified with 1.5% and 2% of moringa leave extract. Ramachandran et al. (2017) also reported that the microbial count of SCJ with a 10% concentration of moringa seed extract and lemon juice was lesser as compared to other samples during 8 days of storage.

### 3.7 Sensory analysis

**FIGURE 3:**
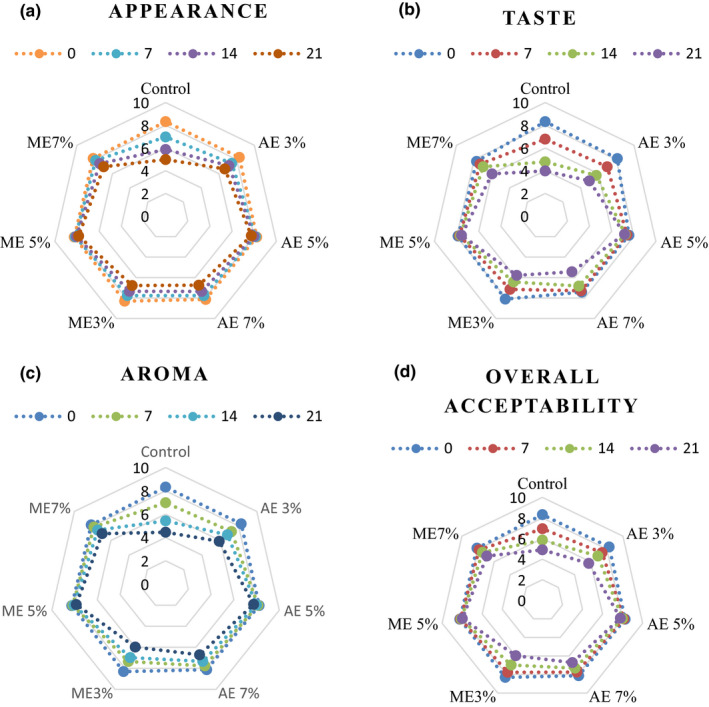
(a–d) Appearance, taste, aroma and overall acceptability score of control and treated SCJ samples during storage

The sensory analysis could be considered as the most important aspect in juices fortified or preserved with natural extracts since it reflects the consumer preferences and acceptability (Yu et al., 2018). Sensory analysis scores of quality attribute such as appearance, taste, aroma, and overall acceptability of control and treated SCJ are presented in Figure 3(a–d). It was observed that all the treated samples were acceptable among different panelists. At zero days of storage, all aonla and moringa treated SCJ samples recorded a slight decrease in sensory parameters as compared to control. This is because the natural extracts can alter the taste of juice due to the stringent flavor associated with its composition (Ajayi et al., 2019). On the other hand, treated samples recorded a high score of sensory attributes during storage as compared to control. However, it could be noticed that there were significant (*p* < .05) differences observed in the appearance, taste, aroma, and overall acceptability among treated SCJ samples. The overall sensory score of AE‐treated SCJ samples significantly decreased (*p* < .05) up to the 21 days of storage while ME‐treated SCJ samples remain acceptable during storage. During storage, control, and both AE and ME (3%) incorporated SCJ deteriorates in appearance, aroma, taste, and overall acceptability because of off‐taste and off‐flavors due to rapid fermentation of sugars. Another reason might be the gradual changes in the physicochemical characteristics, degradation, and oxidation of phytochemicals and microbial growth (Gajera, 2017). It was observed that the treatments with a 5% level of both extracts scored high in all attributes such as appearance, taste, aroma, and overall acceptability. However, the treatments with a 7% level of both extracts scored high in all attributes except taste because the higher level of extract (>5%) might increase the acidity and flavor become stringent. The sensory results are by the findings of Ajayi et al. (2019), who had performed the preservation of zobo juice by adding moringa leave extract at a different level (0.5 and 1%).

## 4 CONCLUSION

The present research demonstrates the preservation of SCJ using natural preservatives that gives more quality and shelf‐life stability in contrast to chemical preservatives. All treatments were studied for physicochemical, nutritional microbial, and sensory analysis at the interval of 7 days during 21 days of storage. ME and AE were observed to help reduce microbial growth and enhance the quality over 21 days. The study concludes that the ME and AE can be effectively used in different proportions as a natural antioxidant and alternative source of chemical preservatives for preserving the quality and inhibition of microbial growth in SCJ. The SCJ juice treated with ME had a more effective stabilizing effect and more nutritional quality as compared to control and AE‐treated SCJ. This can help remove the chemical dependency constraint of SCJ for its preservation and can be used in its commercialization and marketing. This may potentially increase the economic benefits by increasing its round‐the‐year production of sugarcane and its products at the industrial level and its availability in the packed form to meet the consumer demand for healthy and more nutritious refreshing drinks.
